# Cohort Differences in Cognitive Aging in the Longitudinal Aging Study Amsterdam

**DOI:** 10.1093/geronb/gbw129

**Published:** 2016-09-29

**Authors:** Anamaria Brailean, Martijn Huisman, Martin Prince, A Matthew Prina, Dorly J H Deeg, Hannie Comijs

**Affiliations:** 1 Department of Health Service and Population Research, Institute of Psychiatry, Psychology and Neuroscience, Centre for Global Mental Health, King’s College London, UK; 2 Department of Epidemiology and Biostatistics, Amsterdam, The Netherlands; 3 Department of Psychiatry, EMGO Institute for Health and Care Research, VU University Medical Center, Amsterdam, The Netherlands

**Keywords:** Aging, Cognitive abilities, Cognitive reserve, Cohort differences, Education

## Abstract

**Objectives:**

This study aims to examine cohort differences in cognitive performance and rates of change in episodic memory, processing speed, inductive reasoning, and general cognitive performance and to investigate whether these cohort effects may be accounted for by education attainment.

**Method:**

The first cohort (*N* = 705) was born between 1920 and 1930, whereas the second cohort (*N* = 646) was born between 1931 and 1941. Both birth cohorts were aged 65 to 75 years at baseline and were followed up 3 and 6 years later. Data were analyzed using linear mixed models.

**Results:**

The later born cohort had better general cognitive performance, inductive reasoning, and processing speed at baseline, but cohort differences in inductive reasoning and general cognitive performance disappeared after adjusting for education. The later born cohort showed steeper decline in processing speed. Memory decline was steeper in the earlier born cohort but only from Time 1 to Time 3 when the same memory test was administered. Education did not account for cohort differences in cognitive decline.

**Discussion:**

The later born cohort showed better initial performance in certain cognitive abilities, but no better preservation of cognitive abilities overtime compared with the earlier born cohort. These findings carry implications for healthy cognitive aging.

Aging is associated with a general decline in cognitive performance (Brayne et al., 1999; H. L. Park, O’Connell, & Thomson, 2003; Wilson, Beckett, Bennett, Albert, & Evans, 1999), which is especially pronounced for abilities that require effortful processing and high levels of cognitive resources (H. Christensen, 2001; Hedden & Gabrieli, 2004; D. C. Park & Reuter-Lorenz, 2009). When assessed at the same age, later born cohorts tend to outperform earlier born cohorts on cognitive tasks, a finding known as the “Flynn effect” (Flynn, 1987). Growing evidence suggests that cohort improvements in cognitive performance are maintained across the life span (e.g., Bowles, Grimm, & McArdle, 2005; Pietschnig & Voracek, 2015; Rodgers, 1998; Rönnlund & Nilsson, 2009; Skirbekk, Stonawski, Bonsang, & Staudinger, 2013; Trahan, Stuebing, Fletcher, & Hiscock, 2014). In the global context of population aging and related health care concerns, the finding that cognitive performance may get better across generations holds promise for extending the window of healthy and productive aging. A continuation of cohort improvements in cognitive functioning could offset the age-related cognitive decline. This would imply that, despite living longer, later born cohorts would not live in poorer cognitive health compared with earlier born cohorts. (Skirbekk et al., 2013).

Evidence on cohort differences in cognitive aging is mixed and depends on several factors such as the cognitive domains assessed, participants’ age range, the number of years between birth cohorts, and whether studies examined cohort differences in levels or trajectories of cognitive performance. Previous studies that investigated birth cohort differences in level of cognitive performance in late life found better performance in a later born cohort (1926–1948), compared with an earlier born cohort (1900–1925) in memory, verbal, and spatial ability, but not in processing speed at age 67.5 (Finkel, Reynolds, McArdle, & Pedersen, 2007); better performance in the 1914–1948 cohort compared with the 1886–1913 cohort in spatial orientation, word fluency, inductive reasoning, and verbal meaning, but not in numeric ability at age 70 (Gerstorf, Ram, Hoppmann, Willis, & Schaie, 2011); better performance in the 1908–1940 cohort compared with the 1893–1923 cohort in reasoning, spatial orientation, list recall, and test recall, but not in vocabulary at age 74 (Zelinski & Kennison, 2007); better performance in the 1932–1946 cohort compared with the 1910–1924 cohort in list recall, visual recall, and visual learning at age 61–75 (Baxendale, 2010); better performance in logical reasoning and spatial ability in more recent cohorts born in 1901–1902, 1906–1907, and 1930 and measured at age 70 (Karlsson, Thorvaldsson, Skoog, Gudmundsson, & Johansson, 2015); better performance in processing speed, executive function, letter fluency, and category fluency in the 1932–1943 cohort compared with the 1922–1931, 1912–1921, 1902–1911 cohorts aged 65 and older (Dodge, Zhu, Lee, Chang, & Ganguli, 2014); better perceptual speed performance at mean age 75 in the 1925–1948 cohort compared with 1901–1922 cohort (Gerstorf et al., 2015); better performance on the Mini-Mental State Examination (MMSE) and on a composite of five aging-sensitive cognitive tests in the 1915 cohort assessed at age 95 compared with the 1905 cohort assessed at age 93 (Christensen et al., 2013).

Whereas the studies above have consistently reported better levels of cognitive performance in later born cohorts compared with earlier born cohorts, studies that assessed cohort differences in cognitive trajectories reported mixed findings. Finkel and colleagues (2007) found no differences in cognitive decline from age 62 to age 78 in verbal, spatial, memory, and processing speed abilities between the 1926–1948 cohort and the 1900–1925 cohort. Also, Dodge and colleagues (2014) found no differences in rates of change in psychomotor speed and category fluency between the 1932–1941 cohort and the 1922–1931 cohort or the 1912–1921 cohort, as well as no differences in letter fluency between the 1932–1941 cohort and the 1922–1931 cohort aged 65 and older. These findings are in line with the preserved differentiation hypothesis which posits that cohort differences in levels of cognitive performance are similarly preserved across the life span, resulting in similar (i.e., parallel) rates of cognitive decline between cohorts (Salthouse, 2006). A number of studies found evidence for steeper cognitive decline in earlier born cohorts. Dodge and colleagues (2014) found steeper decline in psychomotor speed and category fluency in the 1902–1911 cohort compared with the 1932–1943 cohort, as well as steeper decline in letter fluency in the 1902–1911 and the 1912–1922 cohorts compared with the 1932–1943 cohort, and steeper decline in executive function in the 1922–1931, 1912–1922, and 1902–1911 cohorts compared with the 1932–1943 cohort aged 65 and older. Gerstorf and colleagues (2011) found steeper decline in spatial orientation, inductive reasoning, word fluency, numeric ability, and verbal meaning from age 50 to age 80 in the earlier born cohort (1886–1913) compared with the later born cohort (1914–1948). Also, Zelinski and Kennison (2007) found steeper decline in vocabulary from age 77 to age 86 in the earlier born cohort (1893–1923) compared with the later born cohort (1908–1940). On the contrary, other studies found that later born cohorts showed steeper cognitive decline. Compared with the 1901 cohort, the 1906 and the 1930 cohorts showed steeper decline in spatial ability, and the 1930 cohort showed steeper decline in reasoning ability between age 70 and age 79 (Karlsson et al., 2015). Also, compared with the 1893–1923 cohort, the 1908–1940 cohort showed steeper decline in text and list recall between age 77 and age 86 (Zelinski & Kennison, 2007). These later findings support the differential preservation hypothesis (Salthouse, 2006) which posits that cohort differences in initial levels of cognitive performance are differentially preserved across the life span, leading to different rates of cognitive decline between cohorts.

Given the increase in educational attainment in most countries (including the Netherlands) over the 20th century (Breen, Luijkx, Müller, & Pollak, 2010), and in view of findings suggesting that education increases cognitive reserve (Stern, 2006), education seems a primary candidate able to account for cohort differences in cognitive functioning in late life. Although several studies reported that higher education attainment is associated with better cognitive performance in old age (e.g., Glymour, Kawachi, Jencks, & Berkman, 2008; Schneeweis, Skirbekk, & Winter-Ebmer, 2014; van Hooren et al., 2007), there is little consistent evidence suggesting that aging-related cognitive decline may be moderated by education attainment (for a review, see Lenehan, Summers, Saunders, Summers, & Vickers, 2015). Existing evidence suggests that education does not account or only partially accounts for cohort differences in levels and trajectories of cognitive functioning in late life. Karlsson and colleagues (2015) found that education accounted for cohort differences in levels of performance and rates of decline in spatial ability, but not in reasoning ability. Other studies found that educational attainment did not account for cohort differences in levels of performance and rates of decline in various cognitive abilities (Christensen et al., 2013; Dodge et al., 2014; Gerstorf et al., 2015; Gerstorf et al., 2011).

Our study aims to expand on previous findings by examining cohort differences in cognitive performance and rates of change in immediate recall, delayed recall, inductive reasoning, processing speed, and general cognitive performance. Furthermore, this study aims to examine whether education may account for any observed cohort differences in levels of performance and rates of change in these cognitive abilities.

## Methods

### Participants

Data were used from the Longitudinal Aging Study Amsterdam (Huisman et al., 2011), an ongoing study that focuses on understanding the interplay of physical, emotional, cognitive, and social functioning in late life. Respondents were recruited from three culturally distinct regions in the Netherlands. The first wave of data was collected in 1992–1993 among a sample of respondents aged 55–84 years. Since then measurement cycles were conducted in this sample about every 3 years. In 2002–2003, a first wave of data was collected for another sample of respondents aged 55–64 years. Since then respondents from this sample were also followed up about every 3 years.

The two birth cohorts included in the present study were selected based on an age range between 65 and 75 years at the moment of the baseline assessments. This age range was used to ensure that repeated measures of all cognitive abilities were available and that there was no overlap between birth cohorts across measurement waves. The first cohort included in the present study was born between 1920 and 1930, whereas the second cohort was born between 1931 and 1941. The cycle 1995–1996 was considered the baseline measurement for the first birth cohort (*N* = 705), whereas the cycle 2005–2006 was considered the baseline for the second birth cohort (*N* = 646). For the first cohort, follow-up measurements were conducted in 1998–1999 and 2001–2002. The second cohort was followed up in 2008–2009 and 2011–2012.

### Instruments

General cognitive performance was assessed using the MMSE (Folstein, Folstein, & McHugh, 1975). The instrument is widely used in epidemiological studies to screen for cognitive impairment and to assess general cognitive function/mental status in older adults and shows satisfactory reliability and construct validity (Tombaugh & Mcintyre, 1992). MMSE scores range from 0 to 30 with higher scores indicating better cognitive performance. In our study, we used the scale score based on the maximum score of spelling or subtraction. Because the MMSE score is negatively skewed at all waves, it was transformed (ln[31– MMSE score]) to obtain a near-normal distribution.

Episodic memory was assessed using the 15 Words Test, a Dutch version of the Auditory Verbal Learning Test (Rey, 1964). The procedure started with a verbal presentation of 15 words, which were repeated during three trials, and participants had to report the words they remembered after each trial. The total score of the three trials was used as a measure of immediate recall, and the score could range between 0 and 45. After a distraction period of about 20 minutes, participants were asked to recall the words they had learned. This was used as a measure of delayed recall, and the total score could range between 0 and 15. To avoid learning effects, at the first follow-up participants in both cohorts were administered a different version of the test from the one used at baseline (i.e., they had to memorize a different list of words). At the second follow-up, they received again the same version of the memory test as the one used at baseline.

Information processing speed was assessed using the Coding Task, also known as the Digit-Symbol Substitution subtest of the Wechsler Adult Intelligence Scale (Wechsler, 1987). In the adapted form of the Coding Task used in Longitudinal Aging Study Amsterdam (LASA), participants were shown two rows of characters and have to match the characters from the upper raw with characters from the lower raw using as many combinations as possible. They were asked to name the corresponding character during three trials, each lasting for 1 minute. We used the total score for the three trials, which could range between 0 and 138. Because the original task was adapted to require a verbal rather than a motor response, it is considered that the test measures cognitive speed rather than motor speed processes.

Inductive reasoning was assessed using the Raven Colored Progressive Matrices (Raven, 1995). Participants were presented with a drawing from which a pattern was missing and they had to choose the correct missing pattern from six alternatives. Raven consists originally of three trials, but in LASA only the first and last trials were used. The test shows a progressive increase in difficulty and scale scores range from 0 to 24. Poor performance on this task is considered a good marker of dementia (Gainotti, Parlato, Monteleone, & Carlomagno, 1992).

Whereas for log-transformed MMSE, lower scores reflect better performance, for all other cognitive measures, a higher score reflects better performance. We used age, gender, education attainment, and number of chronic diseases as covariates. We chose to adjust for cohort differences in the number of chronic diseases based on previous findings of LASA showing that the prevalence of chronic diseases increases in the later born cohort (Deeg, van Vliet, Kardaun, & Huisman, 2013) and that chronic diseases predict decline in several domains of cognitive functioning (Comijs et al., 2009). Education attainment was measured as the number of years of schooling. The number of chronic diseases was based on self-reports and included chronic nonspecific lung disease, cardiac disease, peripheral arterial disease, diabetes mellitus, cerebrovascular accident or stroke, osteoarthritis or rheumatoid arthritis, cancer, hypertension, and a maximum of two other diseases. Compared with general practitioner information, the accuracy of self-reports of these diseases was shown to be adequate (Kriegsman, Penninx, van Eijk, Boeke, & Deeg, 1996).

### Statistical Analysis

Linear mixed model analyses were conducted in SPSS (version 22) to examine cohort differences in baseline performance and rates of change in several cognitive abilities. We used maximum likelihood (ML) estimation which calculates parameters using both cases with complete data and cases with partially missing data. The ML estimator deals with missing data under the missing at random (MAR) assumption. When the missing data mechanism is MAR, missing data is assumed to be “noninformative” or “ignorable,” and it can be predicted by variables included in the model (Little & Rubin, 1987). In this case, the estimation of the model parameters in the presence of missing data would be as if data had been complete. The inclusion of several covariates in our models (i.e., age, gender, chronic diseases, and education) helped to improve the accuracy of the estimates of cohort differences in cognitive functioning under the MAR assumption.

Inductive reasoning was only measured at baseline and at the first follow-up, whereas all other cognitive abilities were also measured at the second follow-up. For immediate recall, delayed recall, processing speed, and inductive reasoning, we used raw scores whereas for MMSE, we used log-transformed scores. A first set of models examined cohort differences in baseline cognitive performance adjusting for age, gender, and number of chronic diseases. A second set of models examined cohort differences in baseline cognitive performance adjusting not only for age, gender, and number of chronic diseases, but also for education (measured as years of schooling). A third set of models included an interaction term between time and cohort to examine cohort differences in cognitive change adjusting for age, gender, and number of chronic diseases. Finally, a fourth set of models reexamined cohort differences in cognitive change adjusting not only for age, gender, and number of chronic diseases, but also for education. Significant interaction effects were followed up by stratified analyses by cohort with the aim to examine whether each cohort experienced significant cognitive decline overtime. One set of stratified analyses adjusted only for age, gender, and chronic diseases, and another set of stratified analyses adjusted also for education. For each model, effect sizes were calculated by dividing each estimate by the standard deviation of the outcome.

Sensitivity analyses were conducted to examine whether attrition may bias findings of cohort differences in cognitive decline. We started by examining the missing data patters in each cohort. Second, we compared the reasons for dropout between cohorts. Third, we conducted logistic regression analyses to examine the predictors of dropout (i.e., baseline cognitive performance, age, gender, education, and number of chronic diseases) in each cohort. Fourth, we examined cohort differences in cognitive decline only among study completers (those with observed data at all time points). Fifth, pattern mixture analyses were conducted to examine whether findings of cohort differences in cognitive decline may be affected by specific missing data patterns.

## Results

Descriptive statistics are presented in Table 1, including the number of participants in each cohort who contributed data on each measure at each assessment occasion. Findings from linear mixed models are presented in Table 2. The first set of models examined cohort differences in baseline cognitive performance adjusted for age, gender, and number of chronic diseases, but unadjusted for education. Findings from these models suggest that the later born cohort had statistically significant higher levels of general cognitive performance, inductive reasoning, and processing speed at baseline, whereas no significant cohort differences were found for immediate and delayed recall.

**Table 1.** Descriptive Statistics for Demographic Characteristics and Cognitive Abilities

Birth cohort 1	Baseline			Follow-up			Follow-up		
(1920–1930)	1995–1996			1998–1999			2001–2002		
	(age 65–75 years)			(age 68–78 years)			(age 71–81 years)		
	*N*	Mean	*SD*	*N*	Mean	*SD*	*N*	Mean	*SD*
Age	705	69.8	2.8						
Education	704	9.0	3.2						
Chronic diseases	705	1.6	1.3						
Inductive reasoning	692	18.6	3.7	595	18.1	3.7			
Processing speed	685	76.9	20.2	594	75.9	18.9	487	74.2	19.8
Immediate recall	694	21.3	5.8	593	20.1	5.9	491	21.1	6.5
Delayed recall	694	6.7	2.8	590	6.1	2.9	490	6.5	3.2
General cognitive performance	705	27.6	2.2	618	27.5	2.4	525	27.2	2.6
Birth cohort 2	Baseline			Follow-up			Follow-up		
(1931–1941)	2005–2006			2008–2009			2011–2012		
	(age 65–75 years)			(age 68–78 years)			(age 71–81 years)		
	*N*	Mean	*SD*	*N*	Mean	*SD*	*N*	Mean	*SD*
Age	646	69.5	3.0						
Education	646	10.0	3.4						
Chronic diseases	646	1.9	1.3						
Inductive reasoning	640	19.2	3.4	528	18.8	3.5			
Processing speed	635	81.5	19.5	525	79.3	20.1	431	77.1	19.3
Immediate recall	637	21.1	6.1	523	18.8	5.5	441	21.5	6.1
Delayed recall	635	6.6	2.9	523	5.5	2.7	439	6.6	3.0
General cognitive performance	646	27.9	2.2	554	27.8	2.2	477	27.7	2.4

*Note:* Age and education were measured in years; in the earlier born cohort, 52.3% of participants were women, whereas in the later born cohort 53.7% of participants were women; for inductive reasoning, data were only available at baseline and at the first follow-up.

**Table 2.** Cohort Differences in Baseline Cognitive Performance and Rates of Change

	Models unadjusted for education				Models adjusted for education			
		95% CI				95% CI		
	*B*	Lower bound	Upper bound	Effect size	*B*	Lower bound	Upper bound	Effect size
Cohort differences in cognitive performance at baseline								
MMSE	0.09**	0.02	0.16	0.14	0.03	−0.04	0.09	0.04
Immediate recall	0.27	−0.35	0.89	0.04	0.79*	0.19	1.39	0.13
Delayed recall	0.12	−0.18	0.42	0.04	0.29	−0.01	0.59	0.10
Processing speed	−5.02***	−7.14	−2.90	0.25	−2.54*	−4.51	−0.57	0.13
Inductive reasoning	−0.57**	−0.95	−0.19	0.16	−0.16	−0.52	0.19	0.04
Cohort differences in cognitive change (time by cohort interactions)								
MMSE								
Time (1 vs. 2)	0.01	−0.06	0.08	0.01	0.01	−0.06	0.08	0.01
Time (1 vs. 3)	0.07	−0.01	0.15	0.11	0.07	−0.01	0.14	0.11
Immediate recall								
Time (1 vs. 2)	1.16***	0.55	1.77	0.19	1.17***	0.56	1.77	0.19
Time (1 vs. 3)	−0.65*	−1.30	<−0.01	0.11	−0.62	−1.27	0.02	0.10
Delayed recall								
Time (1 vs. 2)	0.41**	0.12	0.70	0.14	0.41**	0.12	0.70	0.14
Time (1 vs. 3)	−0.32*	−0.64	−0.01	0.11	−0.31*	−0.62	<−0.01	0.11
Processing speed								
Time (1 vs. 2)	1.47*	0.28	2.65	0.07	1.50*	0.32	2.69	0.08
Time (1 vs. 3)	2.71***	1.43	3.99	0.14	2.76***	1.48	4.04	0.14
Inductive reasoning								
Time (1 vs. 2)	−0.06	−0.41	0.30	0.02	−0.04	−0.40	0.31	0.01

*Notes:* CI = confidence interval; MMSE = Mini-Mental State Examination.

MMSE estimates are based on log-transformed scores obtained using the formula (ln[31–MMSE score]), with lower scores reflecting better general cognitive performance. For cohort, the reference category is the later born cohort. For gender, the reference category is female. All models are adjusted for age, gender, and number of chronic diseases at baseline. Effect sizes were calculated by dividing the estimate by the standard deviation of the outcome.

*p < .05. **p < .01. ***p < .001.

A second set of models examined cohort differences in baseline cognitive performance adjusting not only for age, gender, and number of chronic diseases, but also for education. These models suggest that cohort differences in inductive reasoning and general cognitive performance were no longer significant after adjusting for education. However, later born participants continued to show significantly faster processing speed, and they also showed significantly lower levels of immediate recall compared with earlier born participants.

A third set of models examined cohort differences in cognitive change by including an interaction term between cohort and time in the context of adjustment for age, gender, and number of chronic diseases, but not education. The later born cohort showed steeper decline in processing speed overtime. The later born cohort also showed steeper decline in immediate and delayed recall but only between baseline and the first follow-up assessment (when a different word list was administered). Between baseline and the second follow-up assessment (when the same word list was administered), the later born cohort showed shallower decline in immediate and delayed recall compared with the earlier born cohort. We found no significant cohort differences in rates of change in general cognitive performance and inductive reasoning.

**Figure 1.**
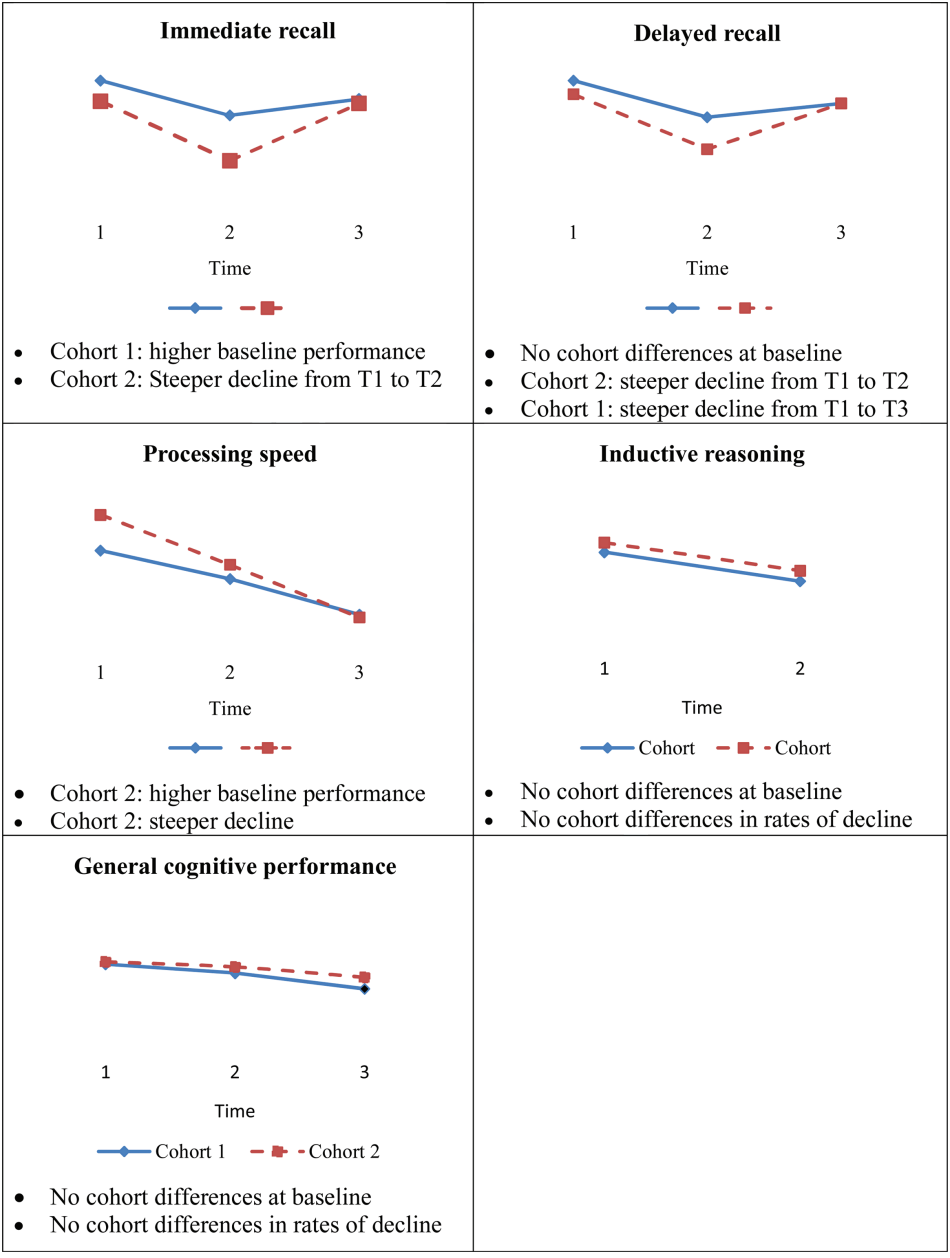
Cohort differences in baseline cognitive performance and rates of decline. Cohort 1 = earlier born cohort; Cohort 2 = later born cohort; for inductive reasoning, data were available only at Time 1 and Time 2; results presented in this figure are based on the fully adjusted models (i.e., controlling for age, gender, chronic diseases, and education).

A fourth set of models examined cohort differences in cognitive change adjusting not only for age, gender, and number of chronic diseases, but also for education. Later born participants continued to show steeper decline in processing speed overtime. Also, later born participants continued to show steeper decline in immediate recall and delayed recall between Time 1 and Time 2, as well as shallower decline in delayed recall between Time 1 and Time 3. We found no significant cohort differences in rates of change in general cognitive performance and inductive reasoning. Significant interaction effects were followed up by stratified analyses by cohort. Because the significance, sign, and magnitude of the interaction effects were similar before and after adjusting for education, we only present stratified results for the fully adjusted models (Supplementary Table 1). Results from stratified analyses suggest that participants in each cohort showed significant decline in processing speed overtime. The earlier born cohort showed significant decline in immediate and delayed recall from Time 1 to Time 2, as well as from Time 1 to Time 3, whereas the later born cohort showed significant decline in immediate and delayed recall only from Time 1 to Time 2. Based on the fourth set of models, we present a figure illustrating the main findings of cohort differences in cognitive functioning (Figure 1), as well as two tables presenting the effects of all covariates (i.e., time, cohort, age, gender, chronic diseases, and education) on cognitive outcomes (Supplementary Tables 2 and 3).

Additional analyses were conducted to examine the robustness of our findings to the effect of attrition. Two main patterns of missing data were identified. Pattern 1 included those who had missing data at both Time 2 and Time 3. Pattern 2 included those who had missing data only at time 3. More than 97% of cases consisted of study completers (i.e., those with observed data at all time points), those with missing data Pattern 1, and those with missing data Pattern 2. Less than 3% of participants had missing data corresponding to a different pattern (e.g., missing only at Time 2). The percentage of participants who completed the study and the percentage of those with specific missing data patterns were similar between cohorts (Supplementary Table 4). In both cohorts, the main reason for dropout was mortality; other reasons included refusal, ineligibility, and lack of contact (Supplementary Table 5). The following predictors of dropout were identified in both cohorts: being male, having a higher number of chronic diseases, and having lower baseline levels of immediate recall, processing speed and inductive reasoning. Older age at baseline and lower levels of delayed recall and general cognitive performance at baseline predicted dropout rates only in the earlier born cohort. Education attainment did not predict dropout rates in either cohort (Supplementary Table 5).

Linear mixed models that included only study completers (Supplementary Table 6) suggest that findings of cohort differences in cognitive decline were similar with findings from the initial analyses that included all participants (i.e., completers as well as dropout cases). The only different finding is that cohort differences in immediate recall from Time 1 to Time 3 failed to reach statistical significance when only study completers were included in the analysis (the effect was marginally significant). Pattern mixture analyses were also conducted to examine whether cohort differences in cognitive decline may vary as a function of the missing data patterns. For the missing data Pattern 1, we calculated cohort by dropout interactions, but it was not possible to examine time by dropout interactions because data were observed only at Time 1. For the missing data Pattern 2 (including observed data at Time 1 and Time 2), we calculated time by cohort by dropout interactions, as well as time by dropout, and cohort by dropout interactions. Time by cohort by dropout (Pattern 2) interactions were not statistically significant, suggesting that changes in cognitive performance overtime in persons with missing data compared with those without missing were similar between cohorts. The only significant interaction between time and dropout (Pattern 2) was found for processing speed (*B* = 2.51, *p* < .05), suggesting that participants who dropped out at Time 3 showed steeper cognitive decline from Time 1 to Time 2 compared with participants who did not dropout at Time 3, regardless of cohort. Cohort by dropout interactions (Pattern 1 and Pattern 2) were not statistically significant, suggesting that cohorts had similar missing data patterns. Adjusting for dropout (Pattern 1 or Pattern 2) did not change findings of cohort differences in cognitive decline (Supplementary Table 7).

## Discussion

Using data from LASA, the present study builds on previous findings of cohort differences in baseline performance and rates of change in various cognitive abilities. In the absence of adjustment for education, the later born cohort showed better general cognitive performance, inductive reasoning, and processing speed, whereas no cohort differences in immediate and delayed recall were found at baseline. After adjustment for education, cohort differences in baseline levels of general cognitive performance and inductive reasoning were no longer found, whereas the later born cohort continued to show faster processing speed. We found no significant cohort differences in rates of change in general cognitive performance and inductive reasoning. However, the later born cohort showed steeper decline in processing speed. The later born cohort also showed steeper decline in immediate and delayed recall but only between baseline and the first follow-up assessment when a different word list was administered. In contrast, the later born cohort showed shallower decline in immediate and delayed recall between baseline and the second follow-up assessment when the same word list was administered. Cohort differences in immediate recall decline between baseline and the second follow-up were no longer found after adjusting for education. Education did not account for cohort differences in cognitive change in any of the other cognitive abilities measured.

The finding that the later born cohort showed better general cognitive performance, processing speed, and inductive reasoning at baseline is consistent with the observation of an increase in cognitive test scores across generations, also known as the “Flynn effect” (Flynn, 1987). The finding that education accounted for cohort differences in general cognitive performance and inductive reasoning is consistent with predictions of the cognitive reserve theory (e.g., Stern, 2002; Stern, 2009). Our finding that education did not account for cohort differences in processing speed is consistent with that of Dodge and colleagues (2014) showing that the 1932–1943 birth cohort had faster processing speed than the 1922–1931 birth cohort aged 65 and older and that the effect persisted after adjustment for education. An unexpected finding is that cohorts showed no differences in memory performance at baseline before adjusting for education, but the earlier born cohort had better immediate recall performance after adjusting for education. This may suggest that older adults in the later born cohort draw upon their higher education to achieve good performance on memory tasks. When adjusting for differences in education attainment between cohorts, the later born cohort no longer benefits from the facilitating effect of education and shows poorer memory performance than the earlier born cohort. The superior memory performance in the earlier born cohort may suggest a shift from rote learning in earlier born cohorts to more meaningful and active learning in later born cohorts (Schaie, 2008), or it may suggest that the memory test administered contains words that are more familiar to earlier born cohorts.

The finding that cohorts showed similar rates of change in general cognitive performance and inductive reasoning provides support for the preserved differentiation hypothesis whereby cohort differences in cognitive performance are similarly preserved overtime, leading to parallel rates of decline in the two cohorts (Salthouse, 2006). However, in line with the differential preservation hypothesis, we found that cohorts showed different rates of decline in processing speed and memory. Steeper decline in processing speed was found in the later born cohort both before and after adjusting for education. These findings are at odds with those of Dodge and colleagues (2014) who found no significant differences in processing speed decline between the 1922–1931 cohort and the 1932–1943 cohort either before or after adjusting for education. Several factors could explain the discrepancy between these findings. First, the study by Dodge and colleagues (2014) included participants who were aged 65 and older at study entry and there was no upper age limit, whereas our study included participants who were aged 65 to 75 years at study entry. Second, whereas the study by Dodge and colleagues (2014) included a task that measured psychomotor speed (i.e., Trail Making Test), our study included an adapted version of the Coding Task which requires a verbal rather than a motor response, thus assessing cognitive speed rather than motor speed processes. Third, the study by Dodge and colleagues (2014) eliminated participants with cognitive impairment (i.e., a score of 21 or below on the MMSE), whereas our study did not select participants based on their level of cognitive functioning.

In interpreting findings of cohort differences in memory decline, it is of note that the same word list was administered to both cohorts at Time 1 and Time 3 and a different word list was administered to both cohorts at Time 2. In both cohorts, we found that decline from Time 1 to Time 2 was steeper than decline from Time 1 to Time 3. This may be due to the greater difficulty of the memory test administered at Time 2, or it may indicate a learning effect between Time 1 and Time 3 when the same memory test was administered. We found that the later born cohort showed steeper decline from Time 1 to Time 2, but shallower decline from Time 1 to Time 3, compared with the earlier born cohort. The steeper decline from Time 1 to Time 2 in the later born cohort may suggest that words presented at Time 2 were less familiar to later born participants, which led to poorer performance in this cohort. It was previously suggested that a drop in the mean difference in education levels between cohorts over assessment waves may cause steeper memory decline in later born participants who lose the advantage of higher education on cognitive function (Zelinski & Kennison, 2007). However, this was not the case in our study. The shallower decline from Time 1 to Time 3 in the later born cohort may suggest that later born participants have better cognitive reserve. Alternatively, this finding may suggest that the later born cohort experiences stronger learning effects between Time 1 and Time 3. However, we believe this is unlikely given the relatively long interval between the first and the third assessment.

We found that education accounted for cohort differences in initial levels of performance in some cognitive domains. These findings may suggest that higher education attainment in later born cohorts may have increased their cognitive reserve, allowing them to tolerate more aging-related neuropathology and maintain better cognitive performance than earlier born cohorts. However, our findings indicate that the later born cohort did not show a superior preservation of cognitive abilities overtime compared with the earlier born cohort, either before or after adjusting for education. These findings may suggest that, once a certain threshold on neuropathological burden is reached and brain reserve/cognitive reserve is exhausted, later born cohorts may experience steeper cognitive decline than earlier born cohorts. In support of these hypothesis, previous studies suggest that cognitive reserve may no longer facilitate cognitive performance once dementia-related neuropathology sets in (Amieva et al., 2014; Hall et al., 2007; Stern, Albert, Tang, & Tsai, 1999). This may explain previous findings suggesting that, despite higher educational attainment, later born cohorts experience steeper terminal cognitive decline (i.e., an acceleration of the rate of cognitive decline before death) compared with earlier born cohorts (Gerstorf et al., 2015; Hülür, Infurna, Ram, & Gerstorf, 2013). Although our study did not directly examine mortality- or dementia-related cognitive decline, our findings suggest that cognitive reserve cannot offset the aging-related brain changes that underlie cognitive decline in community-dwelling older adults. In interpreting current findings, it is of note that our later born cohort had only 1 year of education more than the earlier born cohort. A stronger effect of education on cohort differences in levels and trajectories of cognitive functioning may be observed with larger increases in educational attainment across cohorts. Alternatively, cohort differences in cognitive decline may be better accounted for by factors such as occupational attainment or leisure activities that also contribute to increasing cognitive reserve and delaying cognitive impairment in later life (Scarmeas, Levy, Tang, Manly, & Stern, 2001; Stern, 2012; Stern et al., 1994; Valenzuela & Sachdev, 2006).

A potential limitation of our study is that our findings pertain only to cohorts aged 65 to 75 years at baseline, born 10 years apart, and followed up over 6 years. Future studies should clarify whether our findings can be replicated when longer follow-ups and longer time intervals between birth cohorts are used. Moreover, it remains to examine whether our findings on cohort differences in cognitive abilities in the younger old participants can be replicated in older old persons and whether the protective effect of education on cognitive aging carries on in the last years of life. A common concern in longitudinal studies of aging is the selective dropout of persons with poor physical and cognitive health, which could affect the generalizability of findings. Although we found that persons with poorer baseline cognitive functioning and those with a higher number of chronic diseases were more likely to drop out from the study, the missing data patterns, the reasons for dropout and the predictors of dropout were similar between cohorts. Moreover, complete case analyses and pattern mixture analyses revealed that attrition did not significantly impact on findings of cohort differences in cognitive decline.

To conclude, our findings add to the growing evidence of cohort differences in levels of cognitive performance favoring later born cohorts and suggest that this effect may be partly due to cohort improvements in educational attainment. Our findings suggest that educational attainment may offer later born participants an initial edge in cognitive performance, but it does not slow down their cognitive decline. Understanding the extent to which cohort improvements in cognitive functioning could offset the effect of aging-related cognitive decline has implications for extending the phase of healthy aging and for adapting the workforce and healthcare systems to meet the needs of aging societies.

## Supplementary Material

Please visit the article online at http://psychsocgerontology.oxfordjournals.org/ to view supplementary material.

## Funding

This work was supported by the Marie Curie Initial Training Network project MARATONE (MC ITN-316795 to A. Brailean); Medical Research Council (MR/K021907/1 to A. M. Prina); and The Netherlands Ministry of Health Welfare and Sports, Directorate of Long-Term Care.

## Conflict of Interest

None.

## Supplementary Material

supplementary_materials_JGPS_2015_307_R1
