# Supplementary material for: Cohort Differences in Cognitive Aging in the Longitudinal Aging Study Amsterdam
Source: J Gerontol B Psychol Sci Soc Sci. 2016 Sep 29;73(7):1214–23. doi: 10.1093/geronb/gbw129 (PMC6146762; doi:10.1093/geronb/gbw129)
Supplement: supplementary_materials_JGPS_2015_307_R1 [file gbw129_suppl_supplementary_materials_jgps_2015_307_r1.docx]

**Table 3. Changes overtime in immediate recall, delayed recall and processing speed by cohort**

|  | Mean score | | |  | | 95% CI | | |  |
| --- | --- | --- | --- | --- | --- | --- | --- | --- | --- |
|  | Time 1 | Time 2 | Time 3 | B | Lower bound | | Upper bound | Effect size | |
| **Earlier born cohort** | |  |  |  |  | |  |  | |
| Immediate recall |  |  |  |  |  | |  |  | |
| Time (1 vs. 2) | 21.30 | 19.81 |  | -1.48*** | -1.90 | | -1.07 | 0.25 | |
| Time (1 vs. 3) | 21.30 |  | 20.52 | -0.78** | -1.22 | | -0.33 | 0.13 | |
| Delayed recall |  |  |  |  |  | |  |  | |
| Time (1 vs. 2) | 6.70 | 5.90 |  | -0.80*** | -1.00 | | -0.60 | 0.27 | |
| Time (1 vs. 3) | 6.70 |  | 6.20 | -0.51*** | -0.72 | | -0.29 | 0.17 | |
| Processing speed |  |  |  |  |  | |  |  | |
| Time (1 vs. 2) | 76.78 | 74.71 |  | -2.07*** | -2.88 | | -1.25 | 0.11 | |
| Time (1 vs. 3) | 76.78 |  | 72.28 | -4.49*** | -5.37 | | -3.62 | 0.23 | |
| **Later born cohort** |  |  |  |  |  | |  |  | |
| Immediate recall |  |  |  |  |  | |  |  | |
| Time (1 vs. 2) | 21.10 | 18.43 |  | -2.64*** | -3.08 | | -2.19 | 0.44 | |
| Time (1 vs. 3) | 21.10 |  | 20.94 | -0.13 | -0.61 | | 0.34 | 0.02 | |
| Delayed recall |  |  |  |  |  | |  |  | |
| Time (1 vs. 2) | 6.57 | 5.36 |  | -1.21*** | -1.42 | | -0.99 | 0.42 | |
| Time (1 vs. 3) | 6.57 |  | 6.39 | -0.19 | -0.41 | | 0.04 | 0.07 | |
| Processing speed |  |  |  |  |  | |  |  | |
| Time (1 vs. 2) | 81.71 | 78.15 |  | -3.56*** | -4.43 | | -2.69 | 0.14 | |
| Time (1 vs. 3) | 81.71 |  | 74.47 | -7.24*** | -8.17 | | -6.30 | 0.37 | |

*Note:* * p < 0.05; ** p < 0.01; *** p < 0.001; all models are adjusted for age, gender, number of chronic diseases and education. Results stratified by cohort are presented only for linear mixed models in which time by cohort interaction effects were statistically significant.

Table 4. Effects of cohort, gender, age, chronic diseases, education and time, on general cognitive performance, processing speed and inductive reasoning

| **Model** | **Unadjusted for education** | | |  | **Adjusted for education** | | |
| --- | --- | --- | --- | --- | --- | --- | --- |
|  | B | 95% C.I. | |  | B | 95% C.I. | |
|  |  | Lower Bound | Upper Bound |  |  | Lower Bound | Upper Bound |
| **General cognitive performance** | |  |  |  |  |  |  |
| *Intercept* | -0.63 | -1.31 | 0.05 |  | 0.21 | -0.43 | 0.85 |
| *Main effects* |  |  |  |  |  |  |  |
| Cohort | 0.09** | 0.02 | 0.16 |  | 0.03 | -0.04 | 0.09 |
| Gender | 0.03 | -0.02 | 0.09 |  | 0.12*** | 0.07 | 0.17 |
| Age | 0.02*** | 0.01 | 0.03 |  | 0.02*** | 0.01 | 0.03 |
| Chronic diseases | 0.03** | 0.01 | 0.05 |  | 0.02* | <0.00 | 0.04 |
| Education |  |  |  |  | -0.06*** | -0.07 | -0.05 |
| Time 1 vs. 3 | 0.06* | <0.01 | 0.11 |  | 0.06* | 0.01 | 0.12 |
| Time 1 vs. 2 | 0.03 | -0.02 | 0.09 |  | 0.04 | -0.01 | 0.09 |
| **Processing speed** |  |  |  |  |  |  |  |
| *Intercept* | 156.11*** | 131.15 | 181.08 |  | 121.40*** | 98.36 | 144.45 |
| *Main effects* |  |  |  |  |  |  |  |
| Cohort | -5.01*** | -7.15 | -2.87 |  | -2.52* | -4.50 | -0.54 |
| Gender | -2.81** | -4.87 | -0.76 |  | -6.47*** | -8.39 | -4.56 |
| Age | -1.00*** | -1.36 | -0.64 |  | -0.85*** | -1.18 | -0.52 |
| Chronic diseases | -1.90*** | -2.70 | -1.10 |  | -1.39*** | -2.13 | -0.66 |
| Education |  |  |  |  | 2.49*** | 2.20 | 2.78 |
| Time 1 vs. 3 | -7.20*** | -8.13 | -6.27 |  | -7.25*** | -8.18 | -6.32 |
| Time 1 vs. 2 | -3.54*** | -4.40 | -2.67 |  | -3.57*** | -4.43 | -2.70 |
| **Inductive reasoning** | |  |  |  |  |  |  |
| *Intercept* | 26.62*** | 22.36 | 30.87 |  | 20.99*** | 17.03 | 24.96 |
| *Main effects* |  |  |  |  |  |  |  |
| Cohort | -0.56** | -0.94 | -0.18 |  | -0.17 | -0.53 | 0.19 |
| Gender | 0.67*** | 0.32 | 1.02 |  | 0.07 | -0.26 | 0.40 |
| Age | -0.11** | -0.17 | -0.04 |  | -0.08** | -0.14 | -0.02 |
| Chronic diseases | -0.24** | -0.37 | -0.10 |  | -0.15* | -0.28 | -0.03 |
| Education |  |  |  |  | 0.41*** | 0.36 | 0.46 |
| Time 1 vs. 2 | -0.55*** | -0.81 | -0.30 |  | -0.56*** | -0.82 | -0.30 |

*Note:* * p < 0.05; ** p < 0.01; *** p < 0.001; MMSE estimates are based on log-transformed scores obtained using the formula (ln[31- MMSE score]). Lower log transformed MMSE scores reflect better cognitive performance. For cohort the reference category is the later born cohort. For gender the reference category is female.

Table 5. Effects of cohort, time, gender, age, chronic diseases and education on immediate and delayed recall

| **Model** | **Unadjusted for education** | | |  | **Adjusted for education** | | |
| --- | --- | --- | --- | --- | --- | --- | --- |
|  | B | 95% C.I. | |  | B | 95% C.I. | |
|  |  | Lower Bound | Upper Bound |  |  | Lower Bound | Upper Bound |
| **Immediate recall** |  |  |  |  |  |  |  |
| *Intercept* | 44.75*** | 38.11 | 51.40 |  | 37.83*** | 31.43 | 44.23 |
| *Main effects* |  |  |  |  |  |  |  |
| Cohort | 0.29 | -0.34 | 0.91 |  | 0.79* | 0.19 | 1.40 |
| Gender | -2.68*** | -3.23 | -2.13 |  | -3.40*** | -3.93 | -2.86 |
| Age | -0.32*** | -0.41 | -0.22 |  | -0.29*** | -0.38 | -0.19 |
| Chronic diseases | -0.24* | -0.45 | -0.03 |  | -0.14 | -0.34 | 0.07 |
| Education |  |  |  |  | 0.49*** | 0.41 | 0.57 |
| Time 1 vs. 3 | -0.11 | -0.58 | 0.36 |  | -0.14 | -0.61 | 0.32 |
| Time 1 vs. 2 | -2.63*** | -3.07 | -2.19 |  | -2.64*** | -3.08 | -2.20 |
| **Delayed recall** |  |  |  |  |  |  |  |
| *Intercept* | 15.51*** | 12.27 | 18.74 |  | 13.00*** | 9.81 | 16.19 |
| *Main effects* |  |  |  |  |  |  |  |
| Cohort | 0.15 | -0.15 | 0.45 |  | 0.33* | 0.03 | 0.63 |
| Gender | -1.42*** | -1.68 | -1.15 |  | -1.68*** | -1.94 | -1.41 |
| Age | -0.12*** | -0.16 | -0.07 |  | -0.10*** | -0.15 | -0.06 |
| Chronic diseases | -0.12* | -0.23 | -0.02 |  | -0.09 | -0.19 | 0.01 |
| Education |  |  |  |  | 0.18*** | 0.14 | 0.22 |
| Time 1 vs. 3 | -0.18 | -0.40 | 0.05 |  | -0.19 | -0.42 | 0.04 |
| Time 1 vs. 2 | -1.21*** | -1.42 | -1.00 |  | -1.21*** | -1.42 | -1.00 |

*Note:* * p < 0.05; ** p < 0.01; *** p < 0.001; for cohort the reference category is the later born cohort; for gender the reference category is female.

Table 6. Missing data patterns by cohort

| **Cognitive measure** | **Completers** | **Pattern 1** | **Pattern 2** | **Other patterns** |
| --- | --- | --- | --- | --- |
| Cohort 1 (N = 705) |  | | | |
| Immediate recall | 68% | 14% | 16% | 2% |
| Delayed recall | 68% | 14% | 15% | 3% |
| Processing speed | 67% | 14% | 16% | 3% |
| MMSE | 74% | 12% | 14% | 0% |
| Inductive reasoning | 84% | 14% | N/A | 2% |
| Cohort 2 (N = 646) |  | | | |
| Immediate recall | 64% | 14% | 16% | 3% |
| Delayed recall | 63% | 14% | 17% | 3% |
| Processing speed | 63% | 15% | 18% | 2% |
| MMSE | 72% | 12% | 14% | 2% |
| Inductive reasoning | 81% | 18% | N/A | 1% |

*Note:* Completers are participants with observed data at all time points; pattern 1 consists of observed data at time 1 and missing data at time 2 and time 3; pattern 2 consists of observed data at time 1 and 2 and missing data at time 3.

Table 7. Predictors of dropout and reasons for dropout by cohort

|  | **Earlier born cohort** | | | **Later born cohort** | | |
| --- | --- | --- | --- | --- | --- | --- |
|  | O.R. | 95% C.I. | | O.R. | 95% C.I. | |
|  |  | Lower  bound | Upper  bound |  | Lower  bound | Upper  bound |
| **Predictors of dropout** |  |  |  |  |  |  |
| Age | 1.11** | 1.03 | 1.18 | 1.03 | 0.96 | 1.10 |
| Gender | 0.46*** | 0.31 | 0.68 | 0.60* | 0.40 | 0.89 |
| No. chronic diseases | 1.24** | 1.07 | 1.42 | 1.25** | 1.08 | 1.44 |
| Education | 1.01 | 0.94 | 1.06 | 0.94 | 0.88 | 1.00 |
| Immediate recall | 0.92*** | 0.89 | 0.95 | 0.95** | 0.92 | 0.98 |
| Delayed recall | 0.89** | 0.83 | 0.95 | 0.93 | 0.87 | 1.00 |
| Processing speed | 0.98** | 0.97 | 0.99 | 0.98* | 0.97 | 0.99 |
| Inductive reasoning | 0.93* | 0.89 | 0.98 | 0.92* | 0.87 | 0.98 |
| MMSE | 0.92* | 0.85 | 0.99 | 0.94 | 0.86 | 1.02 |
| **Reasons for dropout** |  |  |  |  |  |  |
| Mortality | 80% |  |  | 57% |  |  |
| Refusal | 12% |  |  | 28% |  |  |
| Ineligibility | 7% |  |  | 14% |  |  |
| Not contacted | 1% |  |  | 1% |  |  |

*Note:* * p < 0.05; ** p < 0.01; *** p < 0.001; all predictors were assessed at baseline;

for gender the reference category is female.

Table 8. Cohort differences in rates of change among study completers

| **Model** | **Unadjusted for education** | | |  | **Adjusted for education** | | |
| --- | --- | --- | --- | --- | --- | --- | --- |
|  |  | 95% CI | |  |  | 95% CI | |
|  | B | Lower bound | Upper bound |  | B | Lower bound | Upper bound |
| Time by cohort interactions | | | | | | |  |
| MMSE |  |  |  |  |  |  |  |
| Time (1 vs. 2) | <-0.01 | -0.08 | 0.07 |  | <-0.01 | -0.08 | 0.08 |
| Time (1 vs. 3) | 0.06 | -0.02 | 0.14 |  | 0.06 | -0.02 | 0.14 |
| Immediate recall | |  |  |  |  |  |  |
| Time (1 vs. 2) | 1.23*** | 0.54 | 1.92 |  | 1.23*** | 0.54 | 1.92 |
| Time (1 vs. 3) | -0.62 | -1.31 | 0.06 |  | -0.62 | -1.31 | 0.06 |
| Delayed recall | |  |  |  |  |  |  |
| Time (1 vs. 2) | 0.44** | 0.11 | 0.78 |  | 0.44** | 0.11 | 0.78 |
| Time (1 vs. 3) | -0.35* | -0.69 | -0.01 |  | -0.35* | -0.69 | -0.01 |
| Processing speed | |  |  |  |  |  |  |
| Time (1 vs. 2) | 1.50* | 0.18 | 2.82 |  | 1.49* | 0.17 | 2.81 |
| Time (1 vs. 3) | 2.91*** | 1.59 | 4.23 |  | 2.90*** | 1.58 | 4.22 |
| Inductive reasoning | |  |  |  |  |  |  |
| Time (1 vs. 2) | -0.04 | -0.40 | 0.32 |  | -0.04 | -0.40 | 0.31 |

*Note:* * p < 0.05; ** p < 0.01; *** p < 0.001; all models were adjusted for age, gender and number of chronic diseases.

Table 9. Cohort differences in rates of change adjusting for dropout patterns

| **Model** | **Unadjusted for education** | | |  | **Adjusted for education** | | |
| --- | --- | --- | --- | --- | --- | --- | --- |
|  |  | 95% CI | |  |  | 95% CI | |
|  | B | Lower bound | Upper bound |  | B | Lower bound | Upper bound |
| Time by cohort interactions  **Pattern 1** | | | | | | |  |
| MMSE |  |  |  |  |  |  |  |
| Time (1 vs. 2) | 0.01 | -0.06 | 0.08 |  | 0.01 | -0.06 | 0.08 |
| Time (1 vs. 3) | 0.07 | -0.01 | 0.15 |  | 0.07 | -0.01 | 0.14 |
| Immediate recall | |  |  |  |  |  |  |
| Time (1 vs. 2) | 1.15*** | 0.54 | 1.76 |  | 1.16*** | 0.55 | 1.76 |
| Time (1 vs. 3) | -0.65* | -1.30 | <-0.01 |  | -0.62 | -1.27 | 0.02 |
| Delayed recall | |  |  |  |  |  |  |
| Time (1 vs. 2) | 0.41** | 0.11 | 0.70 |  | 0.41** | 0.12 | 0.70 |
| Time (1 vs. 3) | -0.32* | -0.63 | -0.01 |  | -0.31* | -0.62 | <-0.01 |
| Processing speed | |  |  |  |  |  |  |
| Time (1 vs. 2) | 1.39* | 0.21 | 2.58 |  | 1.43* | 0.25 | 2.61 |
| Time (1 vs. 3) | 2.69*** | 1.41 | 3.96 |  | 2.74*** | 1.46 | 4.01 |
| Inductive reasoning | |  |  |  |  |  |  |
| Time (1 vs. 2) | -0.04 | -0.39 | 0.31 |  | -0.03 | -0.38 | 0.32 |
| **Pattern 2** | | | | | | |  |
| MMSE |  |  |  |  |  |  |  |
| Time (1 vs. 2) | 0.01 | -0.06 | 0.08 |  | 0.01 | -0.07 | 0.08 |
| Time (1 vs. 3) | 0.07 | -0.01 | 0.14 |  | 0.07 | -0.01 | 0.14 |
| Immediate recall | |  |  |  |  |  |  |
| Time (1 vs. 2) | 1.16*** | 0.55 | 1.77 |  | 1.17*** | 0.56 | 1.77 |
| Time (1 vs. 3) | -0.65* | -1.29 | <-0.01 |  | -0.62 | -1.26 | 0.03 |
| Delayed recall |  |  |  |  |  |  |  |
| Time (1 vs. 2) | 0.41** | 0.12 | 0.70 |  | 0.41** | 0.12 | 0.70 |
| Time (1 vs. 3) | -0.32* | -0.63 | -0.01 |  | -0.31* | -0.62 | <-0.01 |
| Processing speed | |  |  |  |  |  |  |
| Time (1 vs. 2) | 1.47* | 0.28 | 2.66 |  | 1.51* | 0.32 | 2.69 |
| Time (1 vs. 3) | 2.71*** | 1.43 | 4.00 |  | 2.77*** | 1.49 | 4.05 |

*Note:* * p < 0.05; ** p < 0.01; *** p < 0.001; pattern 1 consists of observed data at time 1 and missing data at time 2 and time 3; pattern 2 consists of observed data at time 1 and time 2 and missing data at time 3; all models were adjusted for age, gender and number of chronic diseases.

**Figure 1. Cohort differences in baseline cognitive performance and rates of decline**

| - Cohort 1: higher baseline performance - Cohort 2: Steeper decline from T1 to T2 | - No cohort differences at baseline - Cohort 2: steeper decline from T1 to T2 - Cohort 1: steeper decline from T1 to T3 |
| --- | --- |
| - Cohort 2: higher baseline performance - Cohort 2: steeper decline | - No cohort differences at baseline - No cohort differences in rates of decline |
| - No cohort differences at baseline - No cohort differences in rates of decline |  |

*Note:* cohort 1 = earlier born cohort; cohort 2 = later born cohort; for inductive reasoning data were available only at time 1 and time 2; results presented in this figure are based on the fully adjusted models (i.e., controlling for age, gender, chronic diseases and education).
